# The endoscopic bariatric patient: characteristics, beliefs, and fears

**DOI:** 10.1016/j.igie.2023.12.004

**Published:** 2023-12-23

**Authors:** Daniel B. Maselli, Ashley Kucera, Christopher G. Chapman, Brian Coan, Areebah Waseem, Chase Wooley, Christopher E. McGowan

**Affiliations:** 1 True you Weight loss, Cary, North Carolina, USA; 2 Digestive Diseases and Nutrition, Rush University Medical Center, Chicago, Illinois, USA

## Abstract

**Background and Aims:**

Endoscopic bariatric therapies (EBTs) are minimally invasive tools to facilitate weight loss in adults with obesity and may appeal to those wishing to avoid traditional bariatric surgery (TBS). There are considerable knowledge gaps about patient beliefs, expectations, and concerns about these novel therapies, particularly in comparison with TBS.

**Methods:**

We conducted a 34-question electronic survey of consecutive patients seeking endoscopic sleeve gastroplasty or intragastric balloon placement at a center with expertise in EBT before consultation with a medical provider.

**Results:**

One hundred twenty-five patients were sent surveys, and 101 patients (80.8% response rate, 100% survey completion rate) responded. Patient characteristics were as follows: 88.1% women, mean age 43.2 ± 9.7 years, mean body mass index 38.8 ± 5.6 kg/m^2^, 63.4% white, 32.7% black, 7.9% Hispanic/Latino, 92.1% employed, 58.4% married, and 76.2% had at least 1 obesity-associated comorbidity. In addition, 63.7% of respondents had ≥10 prior weight loss attempts, 64.4% first attempted weight loss before age 26 years, and previous efforts included commercial weight loss programs (66.3%), over-the-counter drugs (66.3%), and prescription antiobesity medications (70.3%). The most common motivations to pursue EBT included desire to lose weight (100%), improve health (98%), improve appearance (87.1%), improve confidence (85.1%), live longer (85.1%), improve mobility (78.0%), and participate in family activities (72.3%). Only 50.5% understood EBTs could have serious adverse events. Sixty-one respondents (60.4%) were eligible for TBS, 18% of whom had met with a bariatric surgeon. Of those eligible for TBS, 67.2% described themselves as either “unlikely” or “extremely unlikely” to pursue TBS; the top reasons cited for preference for EBT over TBS included safety (37.7%), side effects (16.4%), downtime (16.4%), same-day procedure (9.8%), and less preprocedural workup (9.8%).

**Conclusions:**

In this cross-sectional survey of patients seeking EBTs at a practice that focuses solely on nonsurgical management of obesity, patients tended to overestimate efficacy and underestimate the risk of EBTs before undergoing a consultation with a medical provider. This cohort strongly preferred EBT over TBS, primarily driven by perceptions of safety, tolerance, access, and recovery time. This study helps characterize the EBT patient and underscores the importance of patient education in endoscopic treatments of obesity.

Obesity continues to be a significant health problem within the United States and worldwide. To date, the most effective therapy to facilitate weight loss is traditional bariatric surgery (TBS), with nearly 400,000 sleeve gastrectomies and over 200,000 Roux-en-Y gastric bypasses performed annually worldwide.^1^^,^^2^ Nevertheless, less than 1% of patients eligible for TBS pursue this option, primarily attributed to concerns about invasiveness.^3^, ^4^, ^5^ This lack of penetrance represents a critical management gap for patients with obesity who wish to avoid long-term medications or surgical interventions.

Endoscopic bariatric therapies (EBTs) are minimally invasive, nonsurgical, peroral procedures that include space-occupying devices and restrictive gastric remodeling, both of which variably alter peripheral appetite signaling to promote satiety, decrease caloric intake, and facilitate weight loss.^6^, ^7^, ^8^ Popular EBTs in the United States are the intragastric balloon (IGB) and endoscopic sleeve gastroplasty (ESG). IGBs are indicated for treatment of adults with a body mass index (BMI) between 30 and 40 kg/m^2^, whereas the Apollo ESG device (Apollo Endosurgery, Austin, Tex, USA) is authorized by the U.S. Food and Drug Administration for adults with BMIs between 30 and 50 kg/m^2^.^9^^,^^10^ As an emerging technology, EBTs have predictable knowledge gaps about their implementation. A recent survey of primary care providers in an academic center that specializes in EBTs showed that only 47.7% were familiar with EBTs and only 24.6% were familiar with its indications for use.^11^

Awareness of EBT is presumably even more deficient among patients in the early stages of seeking EBT for the treatment of obesity. Although less effective than TBS, EBTs are hypothesized to have greater patient acceptance because of their minimally invasive nature and limited recovery time.^12^ Still, in studies of TBS, patients were found to overestimate risk.^13^ Furthermore, perceived safety profiles may only be one of the reasons a patient may seek an EBT rather than weight loss surgery. The benefit of patient-centered care in enhancing patient outcomes is well documented.^14^^,^^15^ A critical aspect of patient-centered care involves a comprehensive appreciation for patients’ motivations, expectations, and beliefs about a therapeutic approach.^16^^,^^17^ To date, no study has examined patients' attitudes, perceptions, and expectations regarding EBTs or their motivations for pursuing EBTs over TBS.

To enhance our understanding of the type of patients who seek EBT, including their beliefs about EBT, motivations, and factors that influence their preference for EBT over TBS, we surveyed patients with obesity pursuing weight loss through IGB placement or ESG at an ambulatory center specializing in nonsurgical management of obesity.

## Methods

### Study design

This descriptive study intended to assess the motivations of 101 patients who pursued EBT for weight loss using a 1-time point survey. Surveys were completed using a secure online survey platform (Google Forms; Alphabet, Inc, Mountain View, Calif, USA). To assess baseline perceptions and motivations, subjects were provided the survey after they were scheduled for a consultation with our practice’s medical team (physician or nurse practitioner) but before they underwent that consultation. Participants were offered 2 routes of survey administration: by phone with a research coordinator (A.K.) or by e-mail. Information was collected devoid of patient identifiers.

An Institutional Review Board approved the study. All work was conducted in accordance with the Declaration of Helsinki (1964). All patients provided informed consent for their participation in this study.

### Subjects and recruitment

Consecutive adults (age 21-65 years) with a BMI ≥30 kg/m^2^ seeking either IGB placement or ESG at our center from April 1, 2022 to June 6, 2022 and who were willing to complete the study survey either online or by telephone with the help of a research coordinator were included. These subjects were sent information about the opportunity to participate in a survey through e-mail, which explained the purpose of the study, time commitment, and compensation. All subjects who completed their surveys were compensated for their time with an online $50.00 (U.S.$) gift card. Patients were excluded if they had prior IGB therapy because this subset would have been exposed to a previous EBT consultation.

### Reporting and analysis

The 34-question survey is shown in Supplementary Figure 1 (available online at www.igiejournal.org). Responses included discreet selectable answers, free-form numerical responses (eg, age, height, weight), and free responses (eg, “When thinking about endoscopic bariatric therapies, do you have any specific concerns, worries, or fears?”). Responses to questions were kept electronically. Free-form responses were coded to relevant categories.

Responses are reported as frequencies. A subgroup analysis was performed for those seeking ESG-only versus IGB-only therapies as well as in those eligible for TBS based on National Institutes of Health criteria.^18^ Groups were compared on continuous variables using *t* tests and on categorical variables using Fisher exact tests. Results with a *P* ≤ .05 were considered statistically significant.

## Results

### Demographic and anthropometric characteristics

Of 125 consecutive patients seeking either ESG or IGB placement for treatment of primary obesity who were sent surveys, 101 responded. Of the respondents, the survey completion rate was 100%. Demographic and anthropometric characteristics are shown in Table 1.

**Table 1:** Respondent demographic and anthropometric characteristics of entire cohort

Respondent characteristics	Values
No. of female respondents	89 (88.1)
Age, y	
Mean ± standard deviation	43.2 ± 9.7
Median (range)	42 (23-65)
Race	
White, non-Hispanic/Latino	58 (57.4)
Black	31 (30.7)
Hispanic/Latino	8 (7.9)
Mixed race	3 (3.0)
Asian/Pacific Islander	1 (1.0)
Marital status	
Married or domestic partner	59 (58.4)
Single, never married	24 (23.8)
Divorced or separated	16 (15.8)
Widowed	2 (2.0)
Weight, kg	
Mean ± standard deviation	109.5 ± 18.9
Median (range)	110.5 (75.0-158.2)
Body mass index, kg/m^2^	
Mean ± standard deviation	38.8 ± 5.6
Median (range)	38.3 (28.3-55.7)
Obesity class	
I	26 (25.7)
II	34 (33.7)
III	41 (40.6)
Obesity-associated medical problems	
Anxiety/depression	42 (41.6)
Hypertension	33 (32.7)
Dyslipidemia	26 (25.7)
GERD	17 (16.8)
Obstructive sleep apnea	15 (14.9)
Type 2 diabetes mellitus	9 (8.9)
Nonalcoholic fatty liver disease	7 (6.9)

Values are n (%) unless otherwise defined.

Respondents had the following demographic characteristics: 88.1% women, mean age 43.2 ± 9.7 years, mean BMI 38.8 ± 5.6 kg/m^2^, 63.4% white, 32.7% black, 7.9% Hispanic/Latino, 92.1% employed, 58.4% married, and 76.2% had at least 1 obesity-associated comorbidity. When considering EBT options, ESG was the most commonly sought therapy, with 77.2% (n = 78) interested in ESG only, 13.9% (n = 14) interested in IGB only, and the remaining 8.9% (n = 9) interested in either ESG or IGB placement. No differences were found between those seeking only ESG versus those seeking only IGB placement in the proportion of female respondents, excess weight, presence of weight-related medical conditions, or use of prescription antiobesity medications; however, those seeking IGB-only versus those seeking ESG-only therapies had a numerical trend toward younger age (39.6 ± 9.6 years vs 44.2 ± 9.8 years, *P* = .0598) and lower BMI (37.0 ± 4.2 kg/m^2^ vs 39.5 ± 5.7 kg/m^2^, *P* = .0572).

### Weight background, hopes, and expectations

Patient weight loss history, hopes for weight loss, and expectations of clinical effect from EBTs are shown in Table 2. In the overall cohort, most respondents described themselves as “extremely” (n = 51, 50.5%) or “moderately” (n = 32, 31.7%) concerned about their weight. Most respondents had ≥10 prior weight loss attempts (n = 64, 64.4%) and had first attempted weight loss before age 26 years (n = 64, 63.7%). Previous efforts included commercial weight loss programs (n = 67, 66.3%), over-the-counter drugs (n = 67, 66.3%), and prescription antiobesity medications (n = 71, 70.3%). In addition, 64 respondents (63.4%) had been considering a weight loss procedure for less than 2 years. Of the cohort, 31 respondents (30.7%) expected either 30% or 40% total body weight loss, values typically exceeding what is commonly observed after IGB or ESG therapy. Weight loss expectations from EBTs were similar across age groups and classes of obesity represented in this cohort. No differences were found between those seeking ESG-only versus those seeking IGB-only therapies for those features shown in Table 2; however, a numerical trend was found in those considering IGB-only (vs ESG-only) therapy for age of first weight loss attempt under 26 years (85.7% vs 60.3%, *P* = .0778) and for considering a weight loss procedure for less than 1 year (50.0% vs 23.1%, *P* = .0513).

**Table 2:** Respondent weight loss history and perceptions of endoscopic bariatric therapies

Category	Values
No. of prior weight loss attempts	
1-4	5 (5.0)
5-9	28 (27.8)
10-14	24 (23.8)
15-19	8 (7.9)
≥20	36 (35.6)
Age of first weight loss attempt	
≤15 y	20 (19.8)
16-25 y	45 (44.6)
26-35 y	37 (36.6)
36-45 y	8 (7.9)
≥46 y	1 (1.0)
How concerned are you about your weight?	
Extremely concerned	51 (50.5)
Moderately concerned	32 (31.7)
Somewhat concerned	14 (13.9)
Slightly concerned	3 (3.0)
Not at all concerned	1 (1.0)
How much weight are you *hoping* to lose?	
9-18 kg	8 (7.9)
18-27 kg	31 (30.7)
27-36 kg	27 (26.7)
36-45 kg	23 (22.8)
>45 kg	12 (11.9)
If I have an endoscopic weight loss procedure, I will *expect* to lose	
5% of my weight	0 (0)
10% of my weight	0 (0)
15% of my weight	10 (9.9)
20% of my weight	60 (59.4)
30% of my weight	24 (23.8)
40% of my weight	7 (6.9)

Values are n (%).

**Figure 1:**
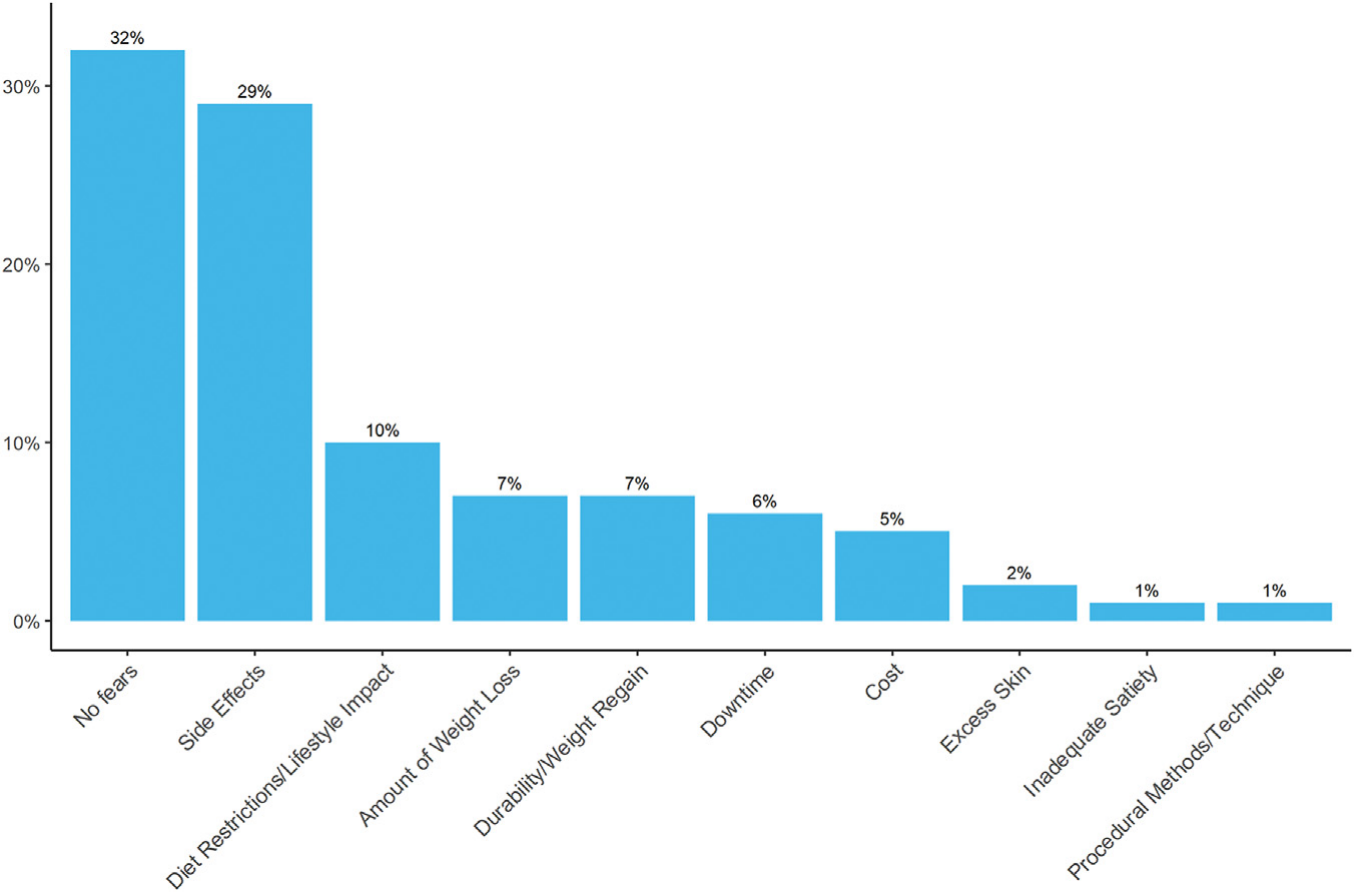
Patient fears and concerns regarding endoscopic bariatric therapies. In a free-form response, patients were asked, “When thinking about endoscopic bariatric therapies, do you have any specific concerns, worries, or fears?” Answers are coded into categories and represented as frequencies.

The most common motivations respondents cited to pursue EBTs are shown in Table 3. Most respondents believed EBTs could improve the following obesity-associated medical problems: hypertension (n = 97, 96.0%), dyslipidemia (n = 97, 96.0%), type 2 diabetes mellitus (n = 95, 94.1%), obstructive sleep apnea (n = 93, 92.1%), and nonalcoholic fatty liver disease (n = 90, 89.1%). In addition, most respondents agreed with the following statements: EBT can be performed outpatient (n = 101, 100%), EBT can be converted to bariatric surgery if needed (n = 76, 75.2%), EBT can be repeated (n = 68, 67.3%), EBT can be reversed (n = 62, 61.4%), and EBT can have serious adverse events (n = 51, 50.5%). Components of an EBT practice valued as “very important” by respondents were physician experience (81.2%), ease of communication with the facility (74.3%), trust in medical staff (73.3%), quality of nutritional support (67.3%), online reputation of the facility (67.3%), quality of psychological support (58.4%), self-pay price (52.4%), and wait time to procedure (45.5%). In a free-response question, patients were asked to list any fears or concerns they had regarding EBT, which were coded into categories shown in Figure 1.

**Table 3:** Most common motivations respondents cited to pursue endoscopic bariatric therapies

Reason for seeking endoscopic bariatric therapies	Affirmative responses
Lose weight	100 (99)
Improve overall health	99 (98)
Improve appearance	88 (87.1)
Improve confidence	86 (85.1)
Live longer	85 (84.2)
Improve mobility	79 (78)
Be able to participate in activities with my family/kids	73 (72.3)
Reduce/stop medications	38 (37.6)
Follow the advice of my primary care provider	14 (13.9)
Reduce the risks from coronavirus disease 2019	7 (6.9)
Prevent comorbidities	2 (2.0)
Improve energy	1 (1.0)
Difficulties with daily activities	1 (1.0)

Values are n (%).

### TBS-eligible subgroup

In this survey of respondents seeking EBTs, 61 of 101 subjects (60.4%) were eligible for TBS. Of these, 41 (67.2%) had BMIs ≥40.0 kg/m^2^ and 20 (32.8%) had BMIs between 35.0 and 39.9 kg/m^2^ with an obesity-associated condition. Mean age was 41.9 ± 4.5 years, mean BMI was 43.5 ± 10.4 kg/m^2^, and mean excess weight was 47.7 ± 12.6 kg. Thirty-eight (62.3%) had a history of prescription antiobesity medication use. Regarding their weight, 54.1% described themselves as “extremely” concerned and 37.7% as “moderately” concerned.

Between those who were eligible versus ineligible for TBS, no statistical differences occurred between the number of female respondents, age, first weight loss attempt under age 26 years, use of prescription antiobesity medications, and percentage pursuing ESG-only therapy; however, a greater proportion described themselves as either “moderately” or “extremely” concerned about weight (91.8% vs 67.5%, *P* = .0029) and number of weight loss attempts ≥10 (80.3% vs 50.0%, *P* = .0021) in those who were eligible versus ineligible for TBS. No statistical differences were seen between those who were eligible and ineligible for TBS among the beliefs shown in Table 4. Of those eligible for TBS, only 11 (18.0%) had met with a bariatric surgeon and 50 (82.0%) had not; however, no statistical differences were found between these groups for the responses shown in Table 4.

**Table 4:** Respondents’ perceptions about EBTs that drive preference for endobariatric therapies over TBSs

Survey statement	Respondents in agreement with statement out of total cohort (n = 101)	Respondents in agreement with statement out of TBS-eligible cohort (n = 61)
EBT can be performed as a same-day procedure	101 (100)	61 (100)
EBT is less invasive than TBS	99 (98.0)	60 (98.4)
EBT requires less downtime from work than TBS	98 (97.0)	59 (96.7)
EBT is safer than TBS	97 (96.0)	60 (98.4)
EBT is more discreet than TBS (no external scars)	96 (95.0)	58 (95.1)
EBT has fewer side effects than TBS	95 (94.1)	56 (91.8)
EBT causes less pain than TBS	95 (94.1)	57 (93.4)
EBT can be obtained more easily/requires less preprocedural workup than TBS	92 (91.1)	57 (93.4)
EBT costs less than TBS	76 (75.2)	44 (72.1)
EBT can be converted to TBS if needed	76 (75.2)	45 (73.8)
EBT can be repeated	68 (67.3)	42 (68.9)
EBT provides the same results as TBS	63 (62.4)	38 (62.3)
EBT can be reversed	62 (61.4)	38 (62.3)
EBT requires less dietary change than TBS	23 (22.8)	11 (18.0)
EBT is the same thing as TBS	14 (13.9)	9 (14.8)

Values are n (%).

*EBT*, Endoscopic bariatric therapy; *TBS*, traditional bariatric surgery.

Of 61 respondents eligible for TBS, overall TBS-related concerns (% of TBS-eligible cohort) were fear of adverse events (62.3%), prolonged recovery or downtime from work (57.4%), cost and lack of insurance coverage (31.1%), fear of death (27.9%), invasiveness (26.2%), poor outcome or adverse event from TBS (24.6%), prolonged evaluation before TBS (13.1%), need for long-term vitamins and supplements (9.8%), fear of pain (6.6%), and stigma (1.6%). The top TBS-related concerns from the TBS-eligible population were fear of surgical adverse events (27.9%), cost and lack of insurance (21.3%), invasiveness (18.0%), prolonged recovery or downtime from work (18.0%), fear of death (9.8%), and religious or cultural reasons (1.6%). Two respondents (3.3%) had no concerns about TBS.

### EBT versus TBS

**Figure 2:**
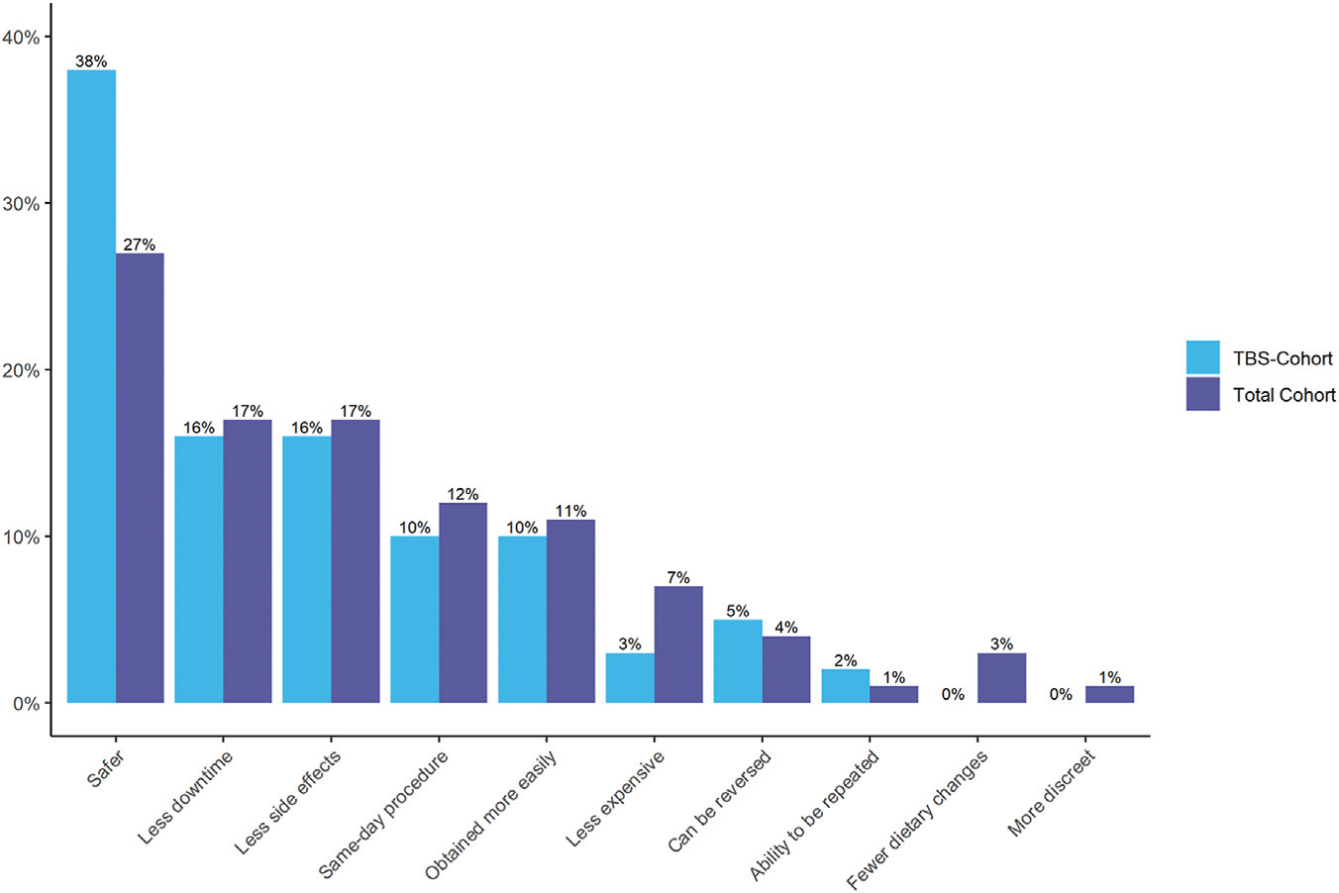
Top reason cited for patient preference for endoscopic bariatric therapies over TBS. Bar graphs represent responses by total cohort and the subset of the cohort eligible for traditional bariatric surgery (TBS-Cohort). *TBS*, Traditional bariatric surgery.

Of 61 respondents seeking EBTs who were eligible for TBS, 52 (85.2%) agreed with the statements that EBTs were either “much better” (n = 33, 54.1%) or “somewhat better” (n = 19, 31.1%) than TBS. Forty-one respondents (67.2%) described themselves as “extremely unlikely” (n = 27, 44.3%) or “unlikely” (n = 14, 23.0%) to pursue TBS. In contrast, 60 respondents (98.4%) described themselves as “very likely” (n = 50, 82.0%) or “likely” (n = 10, 16.4%) to pursue EBT. Of the total cohort and those eligible for TBS, respondents’ most frequently listed perceived advantages of EBT compared with TBS are summarized in Figure 2, with no statistical differences in proportion of affirmative responses between these 2 groups (Table 5).

**Table 5:** Top cited reason for preference for EBT over TBS

Top reason for preference for EBT over TBS	Total cohort (n = 101)	TBS-eligible (n = 61)
Safer than surgery	28 (27.2)	23 (37.7)
Fewer side effects than surgery	17 (16.8)	10 (16.4)
Less downtime from work	17 (16.8)	10 (16.4)
Performed as a same-day procedure	12 (11.9)	6 (9.8)
Obtained more easily (fewer steps beforehand)	11 (10.9)	6 (9.8)
Less expensive	7 (6.9)	2 (3.3)
Reversibility	4 (4.0)	3 (4.9)
Fewer dietary changes	3 (3.0)	0 (0)
Ability to be repeated	1 (1.0)	1 (1.6)
More discreet (no external scars)	1 (1.0)	0 (0)

Values are n (%).

*EBT*, Endoscopic bariatric therapy; *TBS*, traditional bariatric surgery.

## Discussion

This is the first study to examine patients' baseline characteristics, beliefs, and motivations for seeking EBT, especially as they relate to TBS. This sample of patients seeking EBT at our practice was comprised predominantly of women (nearly 9/10) and had a mean age of 43 years. There was representation from class I, II, and III obesity, and over three-fourths had at least 1 obesity-associated medical problem. Over half of the cohort was married, and nearly two-thirds were white. These demographic characteristics mirror those observed in studies of EBT and TBS in the United States.^19^

The cohort assessed in this study is representative of the typical female-to-male ratio of patients seeking EBTs, including large multicenter trials of ESG^10^ and IGB,^20^ as well as proportions observed in the surgical treatment of obesity.^21^ This is congruent with population-based studies about women being more likely to be diagnosed with and to pursue treatment for obesity, likely a result of sex-based differences in the prevalence of obesity, attitudes toward and tolerance of seeking health care, and sociocultural pressures regarding appearance.^22^

Strikingly, most of our cohort had more than 10 prior weight loss attempts, with the first attempt occurring before age 26 years, and 7 of 10 had previously been prescribed weight loss medications. This illustrates and reinforces the updated paradigm of obesity as a complex, chronic, progressive, and relapsing disorder.^23^ When this phenomenon is viewed in the context of our cohort describing moderate or extreme concerns about weight, it is unsurprising that obesity is associated with concomitant psychological distress and decreased quality of life. Implementing EBTs after unsuccessful or nonsustained weight loss from antiobesity medication represents an appropriate escalation of therapy in line with proposed multidisciplinary management strategies.^24^

No major differences were seen in baseline characteristics, weight loss history, and EBT-related beliefs between those seeking ESG-only and those seeking IGB-only therapies, except for numerical trends that showed those seeking IGB placement were modestly younger and had a lower BMI and that a greater proportion had attempted weight loss before age 26 years and had considered an EBT for less than 1 year. We suspect that many differences between these groups were obscured by a low number of participants seeking IGB-only therapy.

In a study by Ahlich et al,^25^ the most common motivations for pursuing surgical weight loss were improving general health (53.9%), improving quality of life (20.7%), and motivations related to mobility and activity engagement with children and family (17.5%), which are similar motivations to other studies of TBS.^5^ Improvement in comorbidity was rated lower (10%). These findings mirror those seen in our study, with priority placed on improved health, quality of life, mobility, and ability to participate in activities with family. Reducing medications (37.6%) and preventing comorbidities (2%) were important but less prioritized. As discussed by Ahlich et al,^25^ although many of these outcomes are downstream effects of weight loss, they merit attention, because focusing purely on weight loss from TBS or IGB placement as the primary motivation limits our full appreciation of the nuances of patients’ goals and may compromise a mission toward patient-centered care.

Given that respondents were drawn from a practice that focuses solely on nonsurgical weight loss, it is perhaps unsurprising that there was a stark bias toward EBT and against TBS, and as such these results cannot be generalized to practices with multidisciplinary approaches that involve endoscopic and surgical management for obesity. In this cohort, greater than 90% of respondents believed, compared with TBS, that EBTs would be less invasive, be safer, require less downtime from work, have fewer side effects, be more discreet, cause less pain, and be obtained more easily. The most prioritized reasons for patients were safety, recovery time, and fewer side effects. Although some of these beliefs may be true at this time (because of a lack of insurance coverage, EBT is not beholden to a prolonged preoperative workup period; the lack of incisions would mean no external scars; and, generally, these are same-day procedures in which return to work can happen in as little as 2-3 days after an outpatient recovery), the safety profile discordance is likely overestimated. This comports with the observation that fear of adverse events from TBS is a top concern for eligible patients, that patients tend to overestimate risk in TBS, and our cohorts’ diminished perception of EBT risk.^5^^,^^13^

The findings from this preconsultation survey identify key targets for patient education. First, nearly one-third expected weight loss of 30% or 40%, which exceeds efficacy typically seen with either ESG or IGB placement, and nearly two-thirds believed EBT would be as effective as TBS. Meta-analysis suggests total body weight loss at 12 months from ESG is approximately 16%.^26^ In our practice, which offers comprehensive, longitudinal aftercare, the mean total body weight loss is approximately 20% at 1 year.^27^ The most effective and increasingly popular EBT for primary obesity, ESG, is still less effective than the most popular TBS, vertical sleeve gastrectomy, after which the ESG was modeled.^28^^,^^29^ This knowledge gap may be because of the relatively recent emergence of EBTs and the lack of a comprehensive patient education curriculum, similar to what has been observed in those considering TBS.^30^ Indeed, patient expectations in TBS also exceed reported weight loss outcomes.^31^^,^^32^ Although weight loss outcomes were overestimated in nearly one-third of respondents, over 90% of respondents correctly believed that the typical degree of weight loss observed with EBTs is associated with improvement in obesity-associated medical problems, highlighting an understanding of weight loss as a force multiplier for improved overall health.^33^

A second target of patient education involves safety. Approximately one-half of the total cohort did not believe that EBTs could have serious adverse events. Although rare, serious adverse events—including GI bleeding, intra-abdominal abscess, GI obstruction, perforation, or GI intolerance and dehydration requiring hospitalization—have been observed with ESG, IGB placement, or both.^34^ Although safety comparisons between ESG and sleeve gastrectomy are mixed within the published literature, with some favoring ESG,^28^ some studies have shown no statistical differences in short-term adverse events between the 2 interventions.^29^^,^^35^

These observations surrounding efficacy and risk of EBT highlight that the former is overestimated and the latter underestimated by a notable portion of those seeking EBT and requires addressing during EBT consultation. Importantly, the responses of this cohort were collected from prospective patients before any consultation with a member of our practice's care team, which comprises physicians, nurse practitioners, and registered dieticians and involves frank discussions about the efficacy, safety, and aftercare involved in EBT. It would be instructive to see how subjects responses changed in an identical postconsultation survey.

Furthermore, these findings also highlight the increased reach of a multidisciplinary approach to obesity as a means of closing the obesity management gap between pharmacologic and surgical obesity therapies.^36^ Over one-half of this cohort was eligible for TBS (and likely more because eligibility has expanded to lower BMI ranges with concomitant diabetes).^18^ However, approximately two-thirds were unlikely to pursue TBS. Although we did not find that consultation with a surgeon statistically changed patient beliefs regarding EBT versus TBS in the domains queried, this was likely because a small percentage of this cohort had seen a bariatric surgeon. From these observations, we draw at least 2 hypotheses. First, patients benefit from increased multidisciplinary consultation irrespective of their expressed preferences, meaning all those eligible for TBS should undergo a consultation to help recalibrate perceptions about invasiveness, risk, efficacy, recovery, and presurgical workup. Second, these results may imply that EBT and TBS are complementary rather than competitive therapeutic options, given that the former may draw from a patient population unwilling and unqualified to pursue the latter. Nevertheless, this can only be inferred after a patient has had suitable information about both options. For instance, because respondents were sampled from an EBT practice, with so few having seen a bariatric surgeon, it is unknown if patients’ lack of acceptance of TBS led to an exaggerated sense of risk from TBS and diminished sense of risk of EBTs (and an analogous closure of efficacy gap between the 2) or if inaccurate beliefs about these effects drove a preference for EBT.

Strengths of this study include a large number of responses and high survey completion rate. In addition, the survey presented here touched on domains shown to be valuable to patients considering weight loss procedures, including emotional (motivations for seeking a procedure), cognitive (beliefs about capabilities of a procedure), and interpersonal or environmental (limitations from obesity on family activities).^37^ Limitations include recruitment from a single center known for expertise in EBT generally and ESG specifically (possibly explaining the patient preference for ESG) and demographic features unique to the practice’s location. Although representative of our practice and with demographic features similar to those seen in TBS publications, this nevertheless remains a convenience sample, and therefore caution should be taken not to apply these results to a population seeking weight loss more generally, particularly among those seeking consultation for both EBT and TBS, as mentioned above. This survey was conducted after semaglutide was authorized by the U. S. Food and Drug Administration for treatment of obesity, but we did not specifically query about a history of incretin-based therapies or patients’ opinions on preference for EBT over incretin-based therapies, which are especially relevant with the emergence of novel and effective medications of this class. Additionally, socioeconomic information was lacking. Although cost was not the top determinative factor for preferring EBT over TBS in the entire cohort (6.9%) or the TBS-eligible cohort (3.3%), self-pay price was cited by 52.4% of the overall cohort for components of an EBT practice listed as “very important.” We therefore suspect that as patients gain insurance coverage for EBT, the overall number of patients pursuing EBT will increase, although perhaps not necessarily the proportion of those TBS-eligible seeking EBT over TBS. Finally, we did not investigate how patients had acquired knowledge about EBT or TBS, which could help address shortcomings or inaccuracies regarding differences in these therapies. Further areas of study include if particular beliefs or motivations are correlated with more significant weight loss or greater duration of weight loss maintenance and how beliefs and perceptions changed after a consultation with a medical professional about the field of EBT.

Patients seeking EBTs from a single U.S. center with expertise in EBTs were more likely to be women, white, middle-aged, married, to have had multiple prior weight loss attempts, to have at least 1 obesity-associated medical problem, and were at least moderately or extremely concerned about their weight. They do so for various motivations, including health-related, cognitive, and interpersonal reasons. In patients seeking EBT at a nonsurgical practice with expertise in EBTs, there can be a preconsultation overestimation of efficacy and safety, and there was a strong preference for EBT over TBS, primarily driven by perceptions of safety, tolerance, access, and recovery time.

## Disclosure

The following authors disclosed financial relationships: D. B. Maselli, C. G. Chapman, C. E. McGowan: Consultant for Apollo Endosurgery. All other authors disclosed no financial relationships.

## Supplementary data

Supplementary Figure 1 Survey with 34 questions administered to prospective patients seeking either endoscopic sleeve gastroplasty or intragastric balloon placement before consultation with a medical provider. Surveys were completed electronically online or over the phone with a research coordinator. *COVID-19*, Coronavirus disease 2019; *NASH*, nonalcoholic steatohepatitis.
