# Supplementary material for: The endoscopic bariatric patient: characteristics, beliefs, and fears
Source: IGIE. 2023 Dec 23;3(1):82–91. doi: 10.1016/j.igie.2023.12.004 (PMC12850792; doi:10.1016/j.igie.2023.12.004)
Supplement: Supplementary Figure 1 — Survey with 34 questions administered to prospective patients seeking either endoscopic sleeve gastroplasty or intragastric balloon placement before consultation with a medical provider. Surveys were completed electronically online or over the phone with a research coordinator. COVID-19, Coronavirus disease 2019; NASH, nonalcoholic steatohepatitis. [file mmc1.pdf]

Supplemental Figure 1: 34-question survey administered to prospective patients seeking either endoscopic sleeve gastroplasty or intragastric balloon prior to consultation with a medical provider

1. What is your current age?
2. What is your sex?
   - Male
   - Female
   - Prefer not to respond
3. What is your gender identity?
   - Man
   - Woman
   - Transgender
   - Non-binary/non-conforming
   - Prefer not to respond
4. What is your current height (in feet and inches)?
5. What is your current weight (lbs)?
6. What is your race (check all that apply)?
   - American Indian or Alaska native
   - Asian
   - Black or African American
   - Native Hawaiian or Other Pacific Islander
   - White
7. What is your ethnicity?
   - Hispanic or Latino or Spanish origin
   - Not Hispanic or Latino or Spanish origin
8. What is your marital status?
   - Single, never married
   - Married or domestic partner
   - Divorced/separated
   - Widowed
9. Are you currently:
   - Employed for wages
   - Self-employed
   - Out of work and looking for work
   - Out of work but not currently looking for work
   - A homemaker
   - A student
   - Military

- Retired
- Unable to work

10. I am concerned about my health as it relates to my weight:
- Not at all concerned
- Slightly concerned
- Somewhat concerned
- Moderately concerned
- Extremely concerned

11. Do you have any of the following medical conditions? (check all that apply):
- Type II Diabetes
- High blood pressure
- High cholesterol
- Obstructive Sleep Apnea
- Fatty liver disease
- Gastroesophageal reflux disease (acid reflux)
- Depression
- Anxiety
- None of the above

12. How many times in the past have you attempted to lose weight through non-surgical efforts?
- 1-4
- 5-9
- 10-14
- 15-19
- 20 or more

13. Which of the following weight loss efforts have you tried in the past (check all that apply):
- Low-carb diet (Keto, Atkins, or other)
- Exercise
- Intermittent fasting
- Commercial weight loss program (weight watchers, Jenny Craig, or similar)
- Physician-prescribed medication (such as phentermine, Qsymia, fen-phen, Contrave, Saxenda, Wegovy, or similar)
- Over the counter weight loss supplements

- Other:

14. Around what age was your first attempt to lose weight?

- 15 or younger
- 16-25 years old
- 26-35 years old
- 36-45 years old
- 46 or older

15. What are the main reasons or factors that have caused you to gain weight? (open ended**)**

16. How much weight, in pounds, are you hoping to lose?

- More than 100 lb
- 80 to 100 lb
- 60 to 80 lb
- 40 to 60 lb
- 20 to 40 lb

17. Have you already seen a surgeon for consideration of bariatric surgery?

- Yes
- No
- Appointment scheduled

18. The reason(s) I am interested in a Weight Loss Procedure (check all that apply):

- To improve my overall health
- To lose weight
- To improve my mobility and/or reduce joint or body pain
- To improve my appearance
- To improve my confidence and feel better about myself
- To live longer
- To be able to participate in activities with my family and/or kids
- To reduce and/or get off of medications I am taking
- To follow the suggestion of my primary care provider
- To lower risk of COVID-19 side effect/complication
- Other:

19. How long have you been considering a Weight Loss Procedure?

- Less than 1 year

- 1-2 years
- 2-3 years
- 3-4 years
- 5 or more

20. Why is now the right time for you to have a Weight Loss Procedure (open ended**)?**

21. Which ENDOSCOPIC weight loss procedure are you interested in:
- Intragastric balloon (weight loss balloon, including Orbera and Spatz3)
- Endoscopic sleeve gastroplasty (ESG)
- Undecided

22. Are you also interested in traditional Bariatric SURGERY – defined as Roux-en-Y gastric bypass, sleeve gastrectomy, or duodenal switch?
- Yes
- No
- Maybe

23. If you answered NO to the question above: Why are you NOT interested in bariatric surgery (check all that apply)?
- I am afraid of surgical complications
- I am afraid of dying
- Cost (it is too expensive)
- My insurance does not cover bariatric surgery
- I do not have health insurance
- I do not qualify for bariatric surgery
- It takes too long to get evaluated and/or approved to have surgery
- I do not want a prolonged recovery or downtime from work
- I am afraid of pain
- I know someone that had a poor experience or complication from surgery
- I prefer a reversible option
- I am afraid people will judge me for having surgery
- Religious or cultural reasons
- I am afraid I will not be able to maintain the weight loss
- I do not want to take vitamins or supplements long-term
- Surgery is too drastic
- Other

24. What is the TOP reason you are NOT interested in BARIATRIC surgery (only check one)?

- I am afraid of surgical complications
- I am afraid of dying
- Cost (it is too expensive)
- My insurance does not cover bariatric surgery
- I do not have health insurance
- I do not qualify for bariatric surgery
- It takes too long to get evaluated and/or approved to have surgery
- I do not want a prolonged recovery or downtime from work
- I am afraid of pain
- I know someone that had a poor experience or complication from surgery
- I prefer a reversible option
- I am afraid people will judge me for having surgery
- Religious or cultural reasons
- I am afraid I will not be able to maintain the weight loss
- I do not want to take vitamins or supplements long-term
- Surgery is too drastic
- Other

25. Do you think ENDOSCOPIC weight loss procedures:

- Require less dietary changes than surgery – Y or N
- Are less invasive than surgery – Y or N
- Can be obtained more easily than surgery (fewer steps beforehand) – Y or N
- Can be reversed – Y or N
- Can be repeated – Y or N
- Can be converted to a bariatric surgery if needed – Y or N
- Cost less than bariatric surgery – Y or N
- Cause less pain than surgery – Y or N
- Have fewer side effects than surgery – Y or N
- Can be done as an outpatient (same-day) – Y or N
- Require less downtime from work – Y or N
- Are safer than surgery – Y or N
- Are more discreet than surgery (i.e. no external scars) – Y or N

26. What is the TOP reason you are interested in a ENDOSCOPIC weight loss procedure (only check one)

- Are the same thing as bariatric surgery
- Provide the same results as surgery
- Require less dietary changes than surgery

- Are less invasive than surgery
- Can be obtained more easily than surgery (fewer steps beforehand)
- Can be reversed
- Can be repeated
- Can be converted to a bariatric surgery if needed
- Cost less than bariatric surgery
- Cause less pain than surgery
- Have fewer side effects than surgery
- Can have serious complications
- Can be done as an outpatient (same-day)
- Require less downtime from work
- Are safer than surgery
- Are more discreet than surgery (i.e. no external scars)
- Can improve diabetes
- Can improve high blood pressure
- Can improve high cholesterol
- Can improve sleep apnea
- Can improve fatty liver disease (NASH)

27. **When thinking about endoscopic bariatric therapies, do you have any specific concerns, worries, or fears (**open ended**)?**

28. **Compared to Bariatric SURGERY, an Endoscopic weight loss procedure is:**
    - Much worse
    - Somewhat worse
    - About the same
    - Somewhat better
    - Much better

29. **How strongly do you agree with the following statements about an Endoscopic weight loss procedure?** (1=strongly disagree, 2=somewhat disagree, 3=neither agree or disagree (neutral), 4=somewhat agree, 5=strongly agree, 6=N/A(not applicable))
    - It will help me maintain a healthy weight
    - It is a last resort for me
    - It will cause a drastic change in my eating habits
    - It will cause a drastic change in my lifestyle
    - It will be effective in helping me reach my goal weight
    - My family approves of me having a weight loss procedure
    - My partner approves of me having a weight loss procedure

- My friends approve of me having a weight loss procedure
- People will judge me if I have a weight loss procedure
- My family and/or friends are more accepting of endoscopic weight loss compared to bariatric surgery

30. When choosing a center for your ENDOSCOPIC weight loss procedure, how important are the following factors: (1=Not important at all, 2=low importance, 3=neither important or not important (indifferent), 4=important, 5=very important)

- The experience of the physician (number of procedures they have performed)
- Relationship with the medical staff and physician (bedside manner / trust)
- Availability and quality of nutrition support
- Availability and quality of psychology support
- Cost of the procedure (self-pay pricing)
- Online reputation and reviews
- Ease of communicating with the facility and staff
- Wait time to have a procedure

31. If I have an Endoscopic weight loss procedure, I will expect to lose (check one):

- More than 100 lb
- 80 to 100 lb
- 60 to 80 lb
- 40 to 60 lb
- 20 to 40 lb

32. If I have an Endoscopic weight loss procedure, I will expect to lose (check one):

- 40% of my weight
- 30% of my weight
- 20% of my weight
- 15% of my weight
- 10% of my weight
- 5% of my weight

33. As of today, how likely are you to pursue traditional Bariatric SURGERY (such as gastric bypass or vertical sleeve gastrectomy)?

- Extremely Unlikely
- Unlikely
- Neutral
- Likely
- Extremely likely

34. As of today, how likely are you to pursue an ENDOSCOPIC weight loss procedure?

- Extremely Unlikely
- Unlikely
- Neutral
- Likely
- Extremely likely
